# Evolutionary conservation of DNA methylation in CpG sites within ultraconserved noncoding elements

**DOI:** 10.1080/15592294.2017.1411447

**Published:** 2018-02-06

**Authors:** Mathia Colwell, Melissa Drown, Kelly Showel, Chelsea Drown, Amanda Palowski, Christopher Faulk

**Affiliations:** Department of Animal Sciences, University of Minnesota, College of Food, Agricultural, and Natural Resource Sciences, Saint Paul, MN, USA

**Keywords:** Epigenetics, DNA Methylation, Phylogenetic Least Squares, CpG Density, Evolution, Varmints, Ultraconserved Noncoding Elements

## Abstract

Ultraconserved noncoding elements (UCNEs) constitute less than 1 Mb of vertebrate genomes and are impervious to accumulating mutations. About 4000 UCNEs exist in vertebrate genomes, each at least 200 nucleotides in length, sharing greater than 95% sequence identity between human and chicken. Despite extreme sequence conservation over 400 million years of vertebrate evolution, we show both ordered interspecies and within-species interindividual variation in DNA methylation in these regions. Here, we surveyed UCNEs with high CpG density in 56 species finding half to be intermediately methylated and the remaining near 0% or 100%. Intermediately methylated UCNEs displayed a greater range of methylation between mouse tissues. In a human population, most UCNEs showed greater variation than the LINE1 transposon, a frequently used epigenetic biomarker. Global methylation was found to be inversely correlated to hydroxymethylation across 60 vertebrates. Within UCNEs, DNA methylation is flexible, conserved between related species, and relaxed from the underlying sequence selection pressure, while remaining heritable through speciation.

## Abbreviations

Nucleotides: (nt)
Ultraconserved noncoding elements: (UCNEs)
Single nucleotide polymorphism: (SNP)
5’ Cytosine DNA methylation: (5mC)
5’ Cytosine DNA hydroxymethylation: (5hmC)

## Introduction

Ultraconserved noncoding elements (UCNEs) are an especially unusual feature of vertebrate genomes. Of the genome, 1.2% codes for proteins and the remaining 98.8% is noncoding sequence. Interspecies comparisons reveal that 8.2% of the human genome is constrained and conserved when compared across mammals [1]. In 2004, Bejerano et al. compared genomes of human, mouse, and rat, finding 481 regions longer than 200 nt sharing 100% sequence identity [2]. These were termed ultraconserved elements (UCEs). Since then, several databases have been developed to catalog highly conserved elements, each using a slightly different definition. In 2013, Dimitriva and Bucher used slightly relaxed criteria to identify elements >200 nt long with >95% identity between human and chicken, and isolated their orthologues in 18 vertebrate species, within intergenic regions or noncoding regions of DNA. These were termed ‘ultraconserved noncoding elements’ [3]. Their database, UCNEbase, lists 4351 UCNEs and features a consistent naming scheme to identify elements across the 18 disparate genomes, along with descriptive statistics of element distribution and synteny maps.

UCNEs are found throughout the genome and are both intergenic and intronic [2]. Most UCNEs are found clustered together near a presumably regulated gene, while some are found in solo. Their enrichment favors transcription factor-rich regions or key developmental loci [4,5]. One notable example is at the *HOXD* gene. Over a 7 kb range, 30 UCNEs are dispersed upstream of *HOXD*, a gene that has been highly conserved over the course of vertebrate evolution [6]. Despite a bias towards developmental genes, their functions remain unknown in most cases, though cancer cells appear to harbor abnormal dosage of UCNEs [7]. A study of four UCNE knockouts in mice revealed viable mice with no obvious changes in phenotype [8]. However, the study was restricted to a single generation of laboratory-kept mice, and no changes in associated gene expression were seen [8]. In human populations, relatively frequent polymorphisms exist in UCNEs, and their derived alleles are frequently homozygous, similarly suggesting dispensability. Additionally, the average level of selection on UCNEs is less than that of essential genes, suggesting that their extreme conservation is independent of a strong functional constraint, at least in the current population [8,9].

UCNEs are conserved within vertebrates, but no orthologues of vertebrate UCNEs are found outside the phylum Chordata. Therefore, UCNEs are considered to have been under extreme selection pressure for 300–400 million years with the inability to tolerate mutations. Surprisingly, using the slowest observed neutral substitution rate in a 3 Gb genome, odds are less than 1 × 10^−22^ that even a single UCNE >200 nt would exist in a vertebrate genome given the average accumulation rate of mutations over 300 million years [10]. Genomes appear to not tolerate segmental duplications that contain UCNEs either, suggesting a dosage compensation function [11]. The degree of conservation within the core region of a UCNE appears to be more stable than the flanking regions of the UCNE and the length of the conserved region of the UCNE is inversely correlated with evolutionary time [12]. In other words, the core of the UCNE is conserved across deep time, where animals of more recent shared ancestry have greater conservation within the flanks. Selection appears to act at the nucleotide sequence level over the entire length of the UCNE.

Between human and chimpanzee, there is an average of 1% sequence divergence; yet, single nucleotide polymorphism (SNP) point mutations increase to 15% at CpG sites [13]. Within vertebrates, the density of CpG sites and GC content varies, although these species encode a similar number of genes [14]. In animals, generally the cytosines of CpG sites are targets for DNA methylation, an epigenetic mark placed on the DNA without changing its sequence [15]. Based on CpG location and tissue type, a cytosine can be methylated (5mC) or hydroxymethylated (5hmC) on the 5th position of the pyrimidine ring, which can serve as a regulator for certain biological processes such as gene expression [16]. These modifications induce more rapid mutation at CpG sites via a deamination pathway, ultimately depleting heavily methylated vertebrate genomes of CpG dinucleotides. Previous work suggests clade-specific differences in global 5mC levels, with fishes and amphibians having a higher percent 5mC than mammals and birds [17]. However, the conservation of the quantity of 5mC and 5hmC content within a single tissue type across vertebrates has yet to be investigated. Since the evolution of gene regulation is increasingly understood as a key mechanism of speciation and divergence, examining the results of selection pressure on the methylome can reveal the interplay of evolution and DNA methylation without confounding differences in underlying sequence [18].

Within an individual, DNA methylation levels change with age and can be influenced by environmental factors, such as ecological stress, toxicant exposure, or nutrition [16,19,20]. Across species, however, DNA methylation level appears to be correlated with lifestyle traits such as body temperature [21,22]. Additionally, evidence suggests DNA methylation is part of the initiation of divergence and maintenance of species boundaries over evolutionary timescales [23,24]. However, there is conflicting evidence about the stability of DNA methylation within conserved regions of the genome. For instance, Zhang et al. found that genes with stable DNA methylation across tissues were more conserved in comparison to genes with fluctuating methylation [25]. This suggests DNA methylation is stable within ancestral regions of DNA within species. On the contrary, Eckhardt et al. found multiple evolutionarily conserved regions to have differential DNA methylation across tissues, suggesting methylation may be more labile in conserved regions [26]. Others have postulated that DNA methylation is conserved over evolutionary time, but the conservation varies at neutrally evolving regions [13,14]. This led us to ask whether the stability of DNA methylation is conserved within UCNEs over evolutionary time.

Here, we investigate the conservation of DNA methylation within UCNEs across 56 vertebrate species in muscle tissue. UCNEs of >200 nt length and >95% sequence identity in vertebrates were selected from UCNEbase. A total of 18 UCNEs were chosen for analysis, comprising 74 CpG sites, based on high CpG density within the database or similarity between gene orthologues. Additionally, we assessed their methylation levels across 6 tissues within one species, mouse, using males and females to identify sex- and tissue-specific methylation. To measure the variability within one species, human, a random control panel of 96 individual human lymphoblastoid cell DNA extracts were compared. We observed clade specific UCNE DNA methylation across the represented vertebrate species along with UCNE specific DNA methylation across all species. Within mouse, tissue- and sex-specific differences were seen, and within humans, interindividual variation in DNA methylation was pronounced. These findings indicate that DNA methylation is decoupled from sequence conservation at ultraconserved noncoding elements. Therefore, selective pressure to maintain DNA sequence identity at UCNEs does not act on the epigenome, resulting in flexibility and clade-specific innovation in DNA methylation independent of underlying sequence conservation.

## Results

### Global DNA methylation in vertebrates

**Figure 1.**
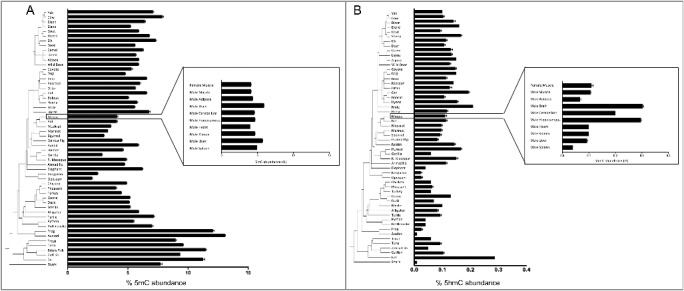
DNA methylation (5mC) and hydroxymethylation (5hmC) content in skeletal muscle of 60 species. (A) 5mC abundance as percentage of total cytosine for muscle tissue and multiple mouse tissues. (B) Percent 5hmC abundance. Shark is designated as the outgroup.

To gain an understanding of the species-specific levels of 5mC and 5hmC present across vertebrates in a single tissue, we first assessed global DNA methylation and hydroxymethylation using mass spectrometry. Values for 5mC and 5hmC are presented in Table 1 and Figure 1. For consistency and to reduce tissue-dependent epigenetic variation, DNA was extracted from skeletal muscle tissue of 60 species representing most classes of vertebrates, ranging from shark to human (Table 1). Fish, amphibians, and reptiles combined average 9.08% 5mC, and 0.075% 5hmC, while birds and mammals have lower average 5mC at 5.2% and higher 5hmC at 0.114%. The two marsupials exhibited the lowest 5mC levels, averaging 2.6%, and low 5hmC at 0.031%. In line with expectations, hydroxymethylation levels in mouse brain regions, whole brain, cerebellum, and hippocampus, averaged higher 5hmC (2.67%) than non-brain tissues (0.082%).

**Table 1.** List of Vertebrate Species and Global DNA Methylation Levels.

Animal Skeletal Muscle	Binomial	Taxon ID	5mC	5mC(se)	5hmC	5hmC(se)
Fish						
Catfish	Unclassified Siluriformes	71179	9.35	0.06	0.10	0.00
Eel	Anguilla Australis	7940	11.25	0.14	0.29	0.00
Mako Shark	Isurus oxyrinchus	57983	7.73	0.11	0.01	0.00
Rainbow Trout	Oncorhynchus mykiss	8022	8.96	0.07	0.06	0.00
Tuna	Scombridae	8233	9.59	0.02	0.09	0.00
Zebrafish	Danio rerio	7955	11.47	0.06	0.05	0.00
Reptiles						
Alligator	Alligator mississippiensis	8496	5.89	0.04	0.08	0.00
Eastern Box Turtle	Terrapene carolina carolina	391345	7.17	0.04	0.09	0.00
Python	Python bivittatus	176946	5.47	0.05	0.04	0.00
Western Diamondback (Rattlesnake)	Crotalus atrox	8730	7.01	0.10	0.04	0.00
Birds						
Chicken	Gallus gallus gallus	208526	4.87	0.05	0.06	0.00
Duck	Anas platyrhynchos	8839	5.13	0.03	0.07	0.00
Goose	Anser sp.	8847	5.15	0.04	0.13	0.00
Merlin	Falco columbarius	8953	5.19	0.03	0.10	0.00
Pheasant	Phasianus colchicus	9054	4.02	0.06	0.06	0.00
Turkey	Meleagris gallopavo	9103	4.46	0.01	0.06	0.00
Amphibia						
Frog	Rana catesbeiana	8400	12.07	0.10	0.03	0.00
Salamander	Ambystoma mexicanum	8296	13.07	0.05	0.01	0.00
Marsupial						
Kangaroo	Macropus sp.	9322	2.49	0.03	0.02	0.00
Opossum	Didelphis virginiana	9267	2.03	0.04	0.03	0.00
Primates						
Gorilla (Western)	Gorilla Gorilla	9593	2.86	0.02	0.06	0.00
Human	Homo sapiens	9609	4.59	0.06	0.17	0.00
Resus Macaque	Macaca mulatta	9544	4.86	0.06	0.15	0.01
Carnivora						
Bear	Ursus americanus	9643	6.54	0.04	0.11	0.00
Bobcat	Lynx rufus	61384	5.03	0.07	0.11	0.00
Cat	Felis catus	9685	6.55	0.04	0.19	0.00
Coyote	Canis latrans	9614	5.30	0.09	0.15	0.00
Dog	Canis lupus familiaris	9615	4.77	0.03	0.15	0.00
Hyena	Crocuta crocuta	9678	5.76	0.06	0.15	0.00
Otter	Lontra canadensis	76717	5.62	0.03	0.13	0.01
Raccoon	Procyon lotor	9654	5.99	0.06	0.14	0.00
Ruminants						
Bison	Bison bison	9901	6.41	0.05	0.14	0.01
Cow	Bos taurus	9913	7.88	0.10	0.10	0.00
Deer	Odocoileus virginianus	9874	5.59	0.01	0.11	0.00
Eland	Taurotragus oryx	9945	5.20	0.03	0.16	0.00
Elk	Cervus canadensis	9864	7.32	0.06	0.11	0.00
Goat	Capra aegagrus hircus	9925	5.89	0.03	0.09	0.00
Sheep	Ovis aries	9940	6.79	0.02	0.17	0.00
Yak	Bos grunniens	30521	7.09	0.07	0.10	0.00
Camelids						
Alpaca	Vicugna pacos	30538	5.91	0.02	0.15	0.00
Camel	Camelus dromedarius	9838	6.25	0.05	0.13	0.01
Llama	Lama glama	9844	5.65	0.03	0.13	0.00
Rodents						
Eastern gray squirrel	Sciurus carolinensis	30640	3.02	0.03	0.09	0.00
Guinea Pig	Cavia porcellus	10141	4.52	0.02	0.08	0.00
Marmot	Marmota monax	9995	3.33	0.01	0.10	0.00
Mouse	Mus musculus	10092	4.10	0.07	0.11	0.01
Muskrat	Ondatra zibethicus	10060	3.60	0.02	0.10	0.00
Rabbit	Oryctolagus cuniculus	9986	5.85	0.06	0.14	0.00
Rat	Rattus norvegicus	10116	4.08	0.05	0.11	0.00
Other						
Armadillo	Dasypodidae	9359	4.73	0.02	0.11	0.00
Eastern Mole	Scalopus aquaticus	71119	5.56	0.04	0.21	0.00
Elephant	Loxodonta africana	9785	6.21	0.06	0.04	0.00
Horse	Equus caballus	9796	6.76	0.09	0.12	0.00
Wild Boar	Sus scrofa subspecies	415978	5.88	0.05	0.13	0.00
**Mouse Tissues**	**Binomial**	**Taxon ID**	**5mC**	**5mC(se)**	**5hmC**	**5hmC(se)**
Mouse Muscle Male	Mus musculus	10092	4.10	0.07	0.11	0.01
Mouse Muscle Female	Mus musculus	10092	4.11	0.02	0.11	0.00
Mouse Adipose Male	Mus musculus	10092	4.35	0.00	0.07	0.00
Mouse Kidney Male	Mus musculus	10092	4.65	0.04	0.10	0.00
Mouse Liver Male	Mus musculus	10092	5.72	0.02	0.09	0.00
Mouse Brain Male	Mus musculus	10092	5.91	0.02	0.30	0.00
Mouse Heart Male	Mus musculus	10092	3.98	0.02	0.10	0.00
Human Lymphoblast	Mus musculus	10092	4.45	0.03	0.04	0.00
Mouse Hippocampus Male	Mus musculus	10092	4.65	0.04	0.30	0.00
Mouse Cerebellum Male	Mus musculus	10092	4.55	0.05	0.20	0.00
Mouse Spleen Male	Mus musculus	10092	4.97	0.03	0.04	0.00

### GC and CpG content

**Figure 2.**
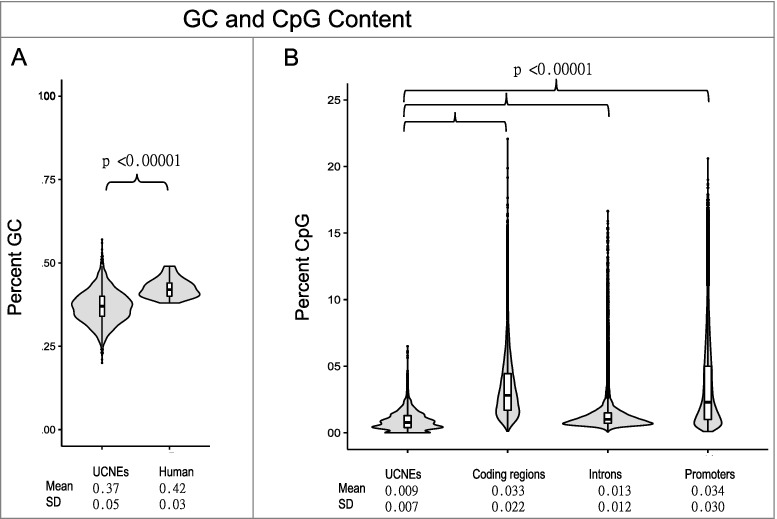
GC and CpG Content in UCNEs. (A) Percent GC of ultra-conserved noncoding elements is lower than the human genome. (B) UCNEs have lower CpG content than coding DNA sequence, introns, or promoters, defined as 1000 nt upstream of coding regions.

The total combined length of the 4351 UCNEs is 693 kb, while their GC content is significantly lower than that of the human genome, 37% vs. 42%, respectively (*P* value = 3.07 × 10^−10^) (Figure 2A). Similarly, the CpG density is lower within UCNEs than in coding sequence, regions 1 kb upstream of genes, and introns (all *P*<0.0001), although the distribution of CpG density most resembles introns (Figure 2B).

### UCNE conservation

A selection of 18 UCNEs with CpG density above 2 sites per 100 nt were chosen to assess for DNA methylation (Table 2). These loci averaged 4.0 CpGs per 100 nt, slightly above the 3.3 CpGs /100 nt seen in coding regions and promoters of the mouse genome, while the average for the complete set of UCNEs was 0.9 CpGs per 100 nt, similar to the 0.8 CpGs per 100 nt global average of the mouse genome. The length of these selected UCNEs was shorter than the overall average, 298 nt vs. 327 nt respectively. All but two loci had a higher than 94% sequence identity between human and the avian zebra finch, and conservation between 62–95% to zebrafish.

**Table 2.** UCNEs queried.

UNCE id	Dataset	Position (GRCh37/hg19)	CpG count	nt	CpG density	# CpGs	Mouse*	Opossum*	Zebra finch*	Zebrafish*
TANK_Chen	a,m	chr2:162095052-162095297	16	246	0.06504065	4	100.00	98.79	95.97	84.23
OTP_Faust	a,m	chr5:76941157-76941363	12	207	0.057971014	4	99.52	98.55	96.14	71.57 to 82.91
PAX9_Emi	a,m	chr14:36741651-36741930	13	280	0.046428571	5	97.86	93.93	95.71	73.45
BCL11B_Xenia	a,m,h	chr14:99738483-99738687	9	205	0.043902439	3	98.54	97.56	97.54	83.33
VRK1_Siddhartha	a,m	chr14:98039411-98039663	11	253	0.043478261	4	97.23	96.05	94.53	81.41
PAX2_Abraham	a,m	chr10:102372689-102372921	10	233	0.042918455	5	100.00	95.71	89.36	89.50
PAX2_Daniel	a,m,h	chr10:102415096-102415542	19	447	0.042505593	4	99.78	98.83	96.85	62.92 to 84.47
SMAD2_Leo	a,m,h	chr18:45121013-45121327	13	315	0.041269841	5	97.77	NA	95.85	84.88
ZNF521_Robert	a,m	chr18:22931939-22932182	10	244	0.040983607	3	100.00	98.74	77.37	88.11
BCL11A_Naoko_2	a,m	chr2:59541513-59541733	9	221	0.040723982	7	99.10	98.19	95.02	NA
chr11_Kaori	a,m	chr11:130106663-130106995	13	333	0.039039039	5	98.76	97.52	96.28	90.31
RNF220_Mateo	a,m	chr1:44990311-44990634	12	324	0.037037037	3	99.07	NA	94.43	70.30
GBX2_Midori	a	chr2:236826292-236826539	9	248	0.036290323	2	98.36	NA	97.53	NA
MICALL2_Mustafa	a,m,h	chr7:1308618-1308845	8	228	0.035087719	3	96.48	96.48	95.13	79.19 to 88.74
HOXD_Malcolm	a,m	chr2:176940301-176940780	15	480	0.03125	4	96.17	98.33	97.08	81.18 to 91.42
MICALL2_Jan	a,m,h	chr7:1278944-1279202	8	259	0.030888031	4	98.07	NA	98.07	68.22 to 95.37
chrX_Maxwell	a	chrX:122599250-122599677	11	428	0.025700935	4	99.26	93.84	95.36	80.10
IRXB_Ruby	a,m	chr16:54323656-54324069	10	414	0.024154589	3	99.03	98.30	97.32	82.72 to 94.59
human LINE1	h	various	NA	NA	NA	4	NA	NA	NA	NA
	a = animal						*Conservation as reported in UCNEbase			
	m = mouse									
	h = human									

### UCNE methylation in vertebrates

**Figure 3.**
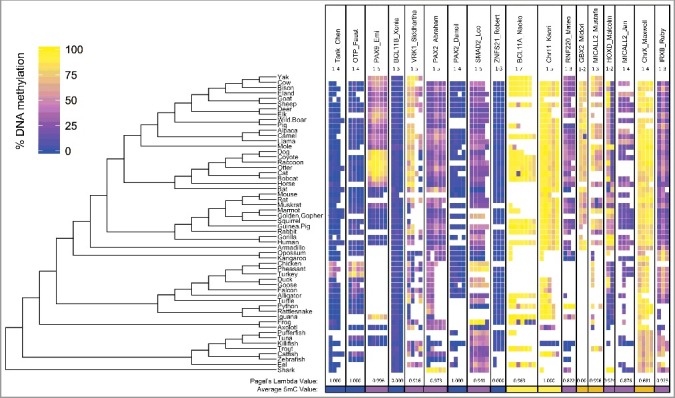
DNA Methylation in UCNEs Across Vertebrates: The DNA methylation of 18 UCNEs are shown for 56 species. UCNEs are listed in order of decreasing CpG density left to right.

A total of 70 CpG sites were assayed in 56 species (Table S2) via pyrosequencing. Of these, 22 sites were below 25% mean DNA methylation across all animals, 16 were above 75%, while most sites (32) were between 25–75% DNA methylation (Figure 3, Table S3). A similar ratio was seen when taking the mean of CpG sites for each UCNE, with 6 UCNEs averaging <25%, 3 at >75%, and 9 intermediate between 25–75%.

In order to determine whether differences in methylation levels corresponded to clade-specific innovations, we used Pagel's lambda (λ) to determine whether there was significant phylogenetic signal. This measure indicates whether traits have evolved similarly due to randomness, or simply because some species are more closely related than others. In 15 of the 18 UCNEs there was high phylogenetic correlation (λ >0.80 and 1 with λ = 0.68) suggesting shared ancestry accounts for similarity in methylation values. For instance, the Phasianidae (ground living) birds are hypermethylated in TANK_Chen and OTP_Faust relative to all other vertebrates. PAX9_Emi reveals a strong phylogenetic signal with Carnivora being highly methylated, and Certeriodactyla (even-toed ungulates and whales) intermediately methylated, and all other vertebrates having near zero methylation with the curious exception of iguana. Laurasiatheria average the highest methylation in VRK1_Siddhartha. The second position of HOXD_Malcolm is hypermethylated in Rodentia, and higher still within primates. Class Aves along with the marsupials are hypermethylated in IRXB_Ruby though it does not rise to significance.

### UCNE methylation in mouse tissues

**Figure 4.**
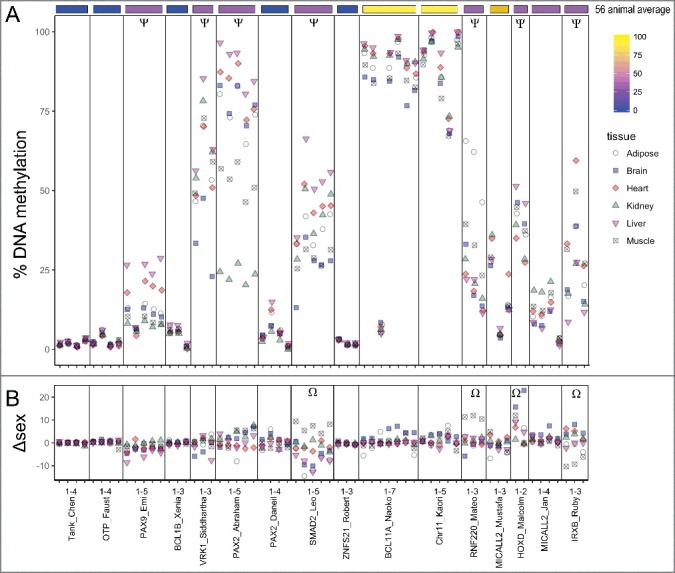
Tissue-specific DNA methylation of UCNEs in Mouse. (A) 16 UCNEs are shown in comparison to the 56 animal average (top). Ψ indicates inter-tissue differences >10% (B) Sex specific differences are shown. Positive values indicate greater methylation in males and negative values for greater methylation in females. Ω indicates inter-sex differences >10%. UCNEs are listed in order of decreasing CpG density left to right.

To compare how tissue origin is reflected in DNA methylation conservation, 16 UCNEs were assessed in 6 different mouse tissues (n = 6 mice): adipose, brain, heart, kidney, liver, and muscle (Figure 4a). Overall, 7 of 16 of the UCNEs showed >10% difference between at least two tissues. For UCNEs where the methylation was in the bottom quartile (below 25%) across species, all tissues in mice also exhibited <25% methylation. Inter-tissue range was also low, with MICALL2_Jan at 8% difference and the rest (all less than 3% range) summarized in Table 3. For two UCNEs in the top quartile across species, all tissues averaged over 75% DNA methylation as well. Inter-tissue range was similarly low with BCL11A_Naoko_2 at 8% and chr11_Kaori at 5% range. UCNEs that were intermediately methylated across vertebrates exhibited the greatest tissue-specific differences within mouse. PAX9_Emi had a range of 15% between kidney and liver (mean 7% and 22%, respectively). VRK1_Siddhartha had a range of 19% (34% in brain to 53% in liver, respectively). PAX2_Abraham exhibited an especially large range of 66% difference between kidney at 24% and liver at 89%. SMAD2_Leo had a range of 26% difference between brain at 26% and liver at 52%. RNF220_Mateo had a range of 40% difference between heart at 18% and adipose at 58%. MICALL2_Mustafa's small range of only 7% difference is lower than vertebrate average methylation with brain at 14% and heart at 21%. HOXD_Malcolm had a range of 18% difference between heart at 31% and liver at 48%. IRXB_Ruby had a range of 24% difference between liver 16% and heart at 40%. In general, over all CpG sites, liver had the highest average methylation and kidney the lowest (Table S3). For individual UCNEs tissue specificity was consistent across CpG sites.

**Table 3.** Mouse Tissue UCNE Methylation.

Mouse tissue	Value	TANK_Chen	OTP_Faust	PAX9_Emi	BCL11B_Xenia	VRK1_Siddhartha	PAX2_Abraham	PAX2_Daniel	SMAD2_Leo	ZNF521_Robert	BCL11A_Naoko_2	chr11_Kaori	RNF220_Mateo	MICALL2_Mustafa	HOXD_Malcolm	MICALL2_Jan	IRXB_Ruby
Adipose	Average	1.78	2.90	11.49	4.88	44.21	74.99	5.51	37.65	2.04	78.67	88.30	58.05	15.44	39.44	10.17	25.20
Brain	Average	1.59	2.83	10.65	3.98	34.64	77.52	4.37	26.19	1.99	73.41	88.31	21.25	14.41	42.87	7.31	24.24
Heart	Average	1.57	1.87	16.45	3.67	44.56	82.13	5.67	43.73	1.97	80.31	90.93	18.11	21.04	31.17	10.09	39.66
Kidney	Average	1.99	2.60	7.49	3.42	50.80	23.48	2.74	41.30	2.11	80.48	88.44	21.70	17.77	33.78	15.22	19.59
Liver	Average	2.46	2.69	22.58	5.78	53.13	89.53	6.48	52.10	2.43	81.37	91.25	18.53	15.89	48.68	7.24	15.98
Muscle	Average	2.29	2.56	8.78	4.55	47.59	53.35	4.38	28.67	2.06	74.93	86.05	31.82	15.76	41.06	11.81	36.09
Adipose	Δ sex (M-F)	0.17	0.25	−2.32	0.66	1.22	−2.45	0.68	−8.37	−0.23	−0.95	1.80	0.48	−3.90	8.18	0.14	−1.24
Brain	Δ sex (M-F)	0.23	0.89	−3.08	0.53	−3.21	2.77	1.55	−5.42	−0.41	3.85	2.28	−1.99	−0.54	19.31	4.08	5.03
Heart	Δ sex (M-F)	0.13	0.57	−2.08	−0.05	0.93	−1.52	0.66	−1.87	−0.31	0.07	0.43	−0.03	2.20	3.08	1.12	3.48
Kidney	Δ sex (M-F)	−0.60	0.01	−0.07	0.04	0.93	3.70	−0.72	−1.05	−0.42	0.30	1.49	1.04	0.29	0.51	1.27	2.34
Liver	Δ sex (M-F)	0.13	0.62	−4.95	−0.50	−2.04	1.24	−0.98	−8.07	−0.71	0.04	0.16	2.24	−2.26	6.95	1.27	−0.28
Muscle	Δ sex (M-F)	−0.34	−0.49	−1.14	−0.60	−0.11	2.95	−1.13	6.89	0.10	0.01	1.51	11.26	−0.12	6.07	0.34	−6.38

To determine whether sex influenced tissue-specific methylation patterns or variability, the difference in mean methylation was calculated for each tissue for 3 mice of each sex (Figure 4b). Overall, 4 of 16 UCNEs showed average inter-sex differences >10%. As with tissue-specific differences, the sex-specific tissue variability corresponded to methylation extremes seen across species. No UCNEs with <25% or >75% methylation across vertebrates had more than 4% methylation difference between sexes for any tissues surveyed. The intermediate UCNEs exhibited sex-specific differences of as little as 3.2% (female vs. male) in brain in VRK1_Siddhartha and up to 19% (male vs. female) in brain in HOXD_Malcolm (Table 3).

### UCNE methylation within humans

**Figure 5.**
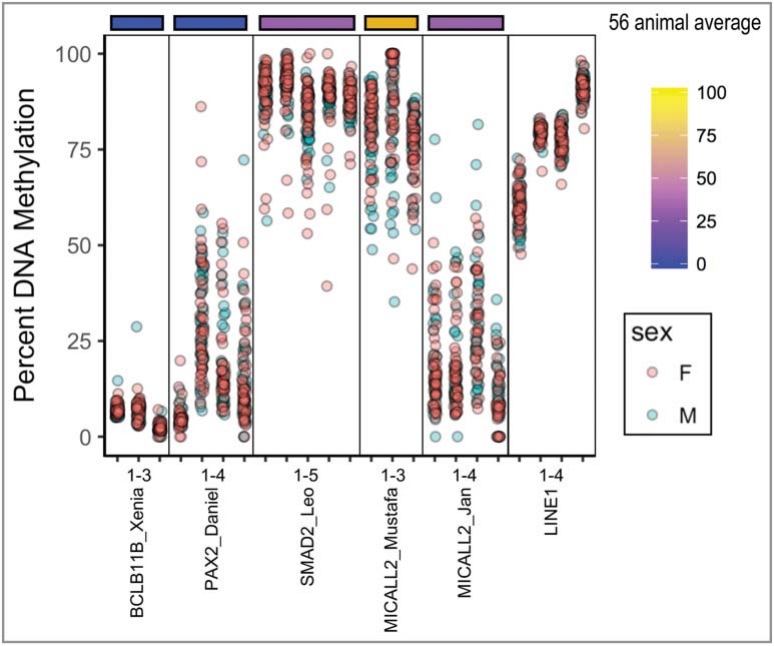
DNA Methylation of UCNEs in a Human population. 5 UCNEs are shown in comparison to the 56 animal average (top). LINE1 element methylation is shown (right). UCNEs are listed in order of decreasing CpG density.

To determine within-species variability, we assessed methylation in lymphoblastoid cell lines derived from 96 human individuals (Figure 5, Table S3). We also compared our lymphoblastoid derived values to the Neandertal and Denosivan values reported by Gokhman et al. from ancient bone [27]. Five UCNEs were chosen representing methylation levels from bottom and intermediate quartiles across vertebrates and compared to the LINE1 transposon family. The LINE1 family serves as a proxy for global methylation as well as being a commonly used biomarker for environmental exposures [28]. BCL11B_Xenia had the lowest methylation (5%) and standard deviation (SD) of 1.5%, similar to its value across vertebrate muscle of 3% (1.7% SD) and 0% in both hominins. In contrast, PAX2_Daniel showed a much higher mean methylation (18%) and variation (9% SD) in humans than in vertebrates [3% (2% SD)] and also averaged 0% in both hominins. SMAD2_Leo had intermediate methylation (33%) and high variation (20% SD) in vertebrates and a much higher methylation level in humans (88%) with low variation (5% SD). Interestingly, Leo's methylation in Neandertal was similar to the vertebrates at 42%, while the Denosivan value of 95% was more similar to the modern human cell line. MICALL2_Mustafa had a similarly high methylation in humans 81% (9% SD), similar to its mean in vertebrates [61% (21% SD)] and similarly high in both hominins, 77% and 100%, respectively. Finally, MICALL2_Jan was 19% (9% SD) in humans and 23% (15% SD) across vertebrates, and discordant in hominins (31% in Neandertal and 0% in Denosivan). For comparison purposes, we assessed LINE1, which showed the expected mean (76% methylation with variation 3%), tighter than all but one UCNE.

## Discussion

Genomes contain a variety of functional elements acting either for the benefit of themselves or the host organism. Genes, promoters, and enhancers adapt for increased host fitness, while transposons selfishly adapt to the host genome and proliferate. UCNEs, however, do not adapt via mutation at all. Their sequence remains the same whether the element occurs within a frog, elephant, whale, or shrew. Such extreme rigidity speaks to an ancient adaptation so strong that no matter how drastic the evolution of the form and function of the host animal, they must remain fixed in sequence. A high level of DNA methylation is also a conserved characteristic of vertebrate animals, as opposed to the mosaic patterns of methylation within invertebrates [29]. However, locus-specific conservation of DNA methylation within ultraconserved noncoding regions across vertebrates has yet to be explored.

In this study, we employed pyrosequencing at 18 CpG rich UCNEs to determine the pattern of methylation conservation using 56 representative species covering 300–400 million years of evolution. We expected DNA methylation at UCNEs to be either near 0% or near 100%, since the overwhelming majority of CpG sites within the genome exhibit one of these maxima [30]. Unexpectedly, we found that several UCNEs exhibited intermediate DNA methylation consistently across their embedded CpG sites. In addition, several UCNEs exhibited clade specific DNA methylation across vertebrates along with sex and tissue specific methylation.

The UCNEs chosen for study here are outliers in having especially high CpG density. The presence of CpGs within them is evidence that while deamination of C to T mutations have likely taken place at the usual rate in the genome, selection pressure has preserved these presumably ancient CpGs just as strongly as non-CpG dinucleotides within the UCNE regions. We highlight this fact since the typical UCNEs has not escaped the rapid loss of CpG sites typically seen throughout vertebrate genomes. Therefore, CpG sites within our chosen CpG rich UCNEs are likely also under selection for their epigenetic function.

### Global DNA methylation and hydroxymethylation of vertebrates

We first ascertained the level of global 5mC methylation and 5hmC hydroxymethylation within the skeletal tissues of the 60 different vertebrate species. The most species-inclusive early measure of global methylation using mass spectrometry by Jabbari et al. used multiple tissues and may be confounded by known tissue-specific effects [17]. However, in agreement with Jabbari et al., we also found that fish, amphibians, and reptiles had higher 5mC in general than birds and mammals when using solely skeletal muscle. The two marsupials measured here had much lower 5mC and 5hmC levels than the rest of the mammals, unsurprising in light of previous research finding that opossums have lower genome-wide CGI density than eutherian mammals [22]. We first report the inverse relationship of 5mC and 5hmC between these groups, with fish, amphibians, and reptiles having lower levels of 5hmC than birds and mammals. Within mouse tissues, the 5hmC results were as expected, with high 5hmC in nervous tissue, and similar to low 5hmc values in other tissues [31].

### UCNE methylation is relaxed in vertebrates

The conservation of DNA methylation within UCNEs is relaxed relative to their extreme sequence conservation. Of the 18 loci selected, most were chosen for having high CpG density when compared to other regions of the genome, although, in general, UCNEs have lower CpG density than other regions (Figure 2). We see relatively large changes between phyla, and detect a significant phylogenetic signal, measured by Pagel's lambda, revealing the covariance among methylation and relatedness. This measure indicates shared ancestry accounts for the shared pattern of conserved DNA methylation within clades. Several UCNEs were elected based on shared characteristics. For example, BCL11A and BCL11B are both highly enriched in CpG sites and share some sequence homology. They reside on different chromosomes and do not share intron-exon structure, although they are both Kruppel-type C2H2 zinc-finger transcription factors that both are required for lymphoid development [32]. Interestingly, their associated UCNEs’ methylation levels are quite divergent (4% vs. 88%). Two pairs of UCNEs were specifically chosen as proximal to the same genes, each of MICALL2 and PAX2, to determine whether methylation was conserved for separate UCNEs regulating the same gene. Neither pair showed similar within-gene methylation patterns. One UCNE was chosen for association with a sex chromosome in humans, chrX_Maxwell, which is intronic to a glutamate receptor (*GRIA3*). Only two UCNEs are found on chromosome Y and neither were assessed, as the sex of most species used here were unknown. Despite a presumed random sex distribution, the highly methylated results for chrX_Maxwell are more consistent with conservation of methylation by clade than sex, since we see a lower methylation value in all fish as compared to the remaining vertebrates. Notably, this is the only UCNE with an intermediate lambda value. While we assessed only a single UCNE near the developmentally crucial HOXD gene several UCNEs exist proximal to it, yet previous research has also discovered at least one differentially methylated region (DMR) near HOXD when comparing human, Denosivan, and Neandertal epigenomes [33], speculated to underlie some of the species specific differences in limb development.

The general pattern of vertebrate DNA methylation has been conserved as evidenced by the epigenome of the basal vertebrate elephant shark [29]. The hypomethylated state of CGIs can be surprisingly stable over evolution, as experimentally verified in previous research across zebrafish, lizard, platypus, chicken, mouse, and human [23]. The hypomethylated UCNEs may benefit from this stability, however, this phenomenon cannot explain the maintenance of CpG sites in heavily methylated UCNEs, since they would be expected to undergo rapid mutation. Conservation of epigenetic marks across species is a defining feature of regulatory regions and methylation status can be closely predicted by CpG density alone [34]. Our previous study found that density of a genomic feature can sufficiently drive selection independent of conservation and other features [35]. Therefore, hypomethylation at UCNEs fits with their putative role as regulatory regions, and hypermethylation may also be under selective pressure independently of underlying sequence [36]. It is less clear why some UCNEs would be intermediately methylated.

Methylation status within UCNEs may be informative from an evolutionary epigenetic standpoint. For instance, Skinner et al. showed 97% of regions with transgenerational epimutations had less than 10% CpG density [37]. We find that UCNEs have less than 6% CpG density and retain methylation states that are clearly heritable by clade, apparently persisting for millions of years.

### Tissue and sex-specific methylation in mice

Differential DNA methylation was seen in several UCNEs between tissues and sexes of biological mouse replicates (Figure 4). While tissue specific methylation is commonly seen within gene promoters, gene bodies, and orthologous genes [38], we showed tissue divergent methylation in 7 of 16 UCNEs within mice. Correlation of highly and lowly methylated UCNEs reflected similar methylation values within mouse tissues as across species in muscle, and loci intermediately methylated across species were the most variably methylated UCNEs in mouse tissues. Strikingly, 4 out of 16 UCNEs exhibited sex-specific methylation patterns. Some regions of the genome are known to have sex-specific methylation that can shift based on environmental stress [39]. Sex-specific differential methylation appears to be decoupled from overall or tissue-specific methylation level. The significance of the sex-specific marks remains unknown.

### Population variation of DNA methylation in UCNEs in humans

Human populations appear to have relaxed stringency at UCEs resulting in increased sequence variation; yet, DNA methylation variability has not been previously tested [40]. To determine within-population variability, we used a human DNA panel of lymphoblastoid cell lines derived from 96 phenotypically normal individuals. There is some doubt about the reliability of epigenetic biomarkers as maintained in immortalized cell lines. However, previous research has validated the use of lymphoblastoid cells as maintaining epigenetic differentiation that matches primary cells [41]. Here, we found that variation across vertebrates was predictive of variability within a population of humans. Interestingly, however, PAX2_Daniel was far higher in humans than all mouse tissues and vertebrates in general, with high interindividual variation. Since methylation of the LINE1 transposon is a frequent biomarker of exposure [42], which relies upon its inherent ability to be variably methylated, we assessed its variability as compared to UCNEs. We found that all but one UCNE had larger interindividual variability than LINE1 [43,44], despite their much greater relative sequence conservation.

Comparison of methylation levels from Neandertal and Denosivan bone also reflect the levels seen in humans at the UCNEs with some intriguing discordances. Smad2_Leo was discordant between average vertebrate level (33%) and human lymphoblastoid results (88%). Interestingly, the Neandertal and Denosivan values are also widely discordant at this locus, 42% and 95%, respectively, although both are derived from bone [27]. So, this UCNE appears to harbor genuine species-specific methylation independent of tissue origin.

### Conclusion

Here, we characterize the DNA methylation characteristics of CpG-rich UCNEs. While other studies have compared methylomes from distantly related pairs of species, we are the first to comprehensively survey vertebrates. UCNEs are currently used in phylogenetic studies to determine deep phylogenetic relationships as well as classifying museum specimens [45,46]. The use of UCNEs can now be expanded as a tool to understand evolutionary epigenetics. The initial discovery and very existence of UCNEs was surprising, and their function remains mysterious. Our results reveal unexpected findings of intermediate methylation, tissue- and sex-specific variation, and wide variability of methylation within humans. Therefore, UCNEs continue to surprise. Due to their ease of amplification across species and their malleability, we encourage their use as potential epigenetic biomarkers. With further research, the methylation status of UCNEs may lead to a better understanding of their functions within the genome.

## Materials and methods

### Tissue collection and DNA extraction

Muscle tissue was sourced through commercial retailers or through gifts from research labs for 60 species (Table S2). As such, no animal ethics permissions were required for these tissues. Mouse tissues were derived from adult (8-12 week old) Agouti viable yellow *A^vy^* strain wild type mice that are 93% homologous to the C57bl/6j strain [47]. DNA was extracted via standard phenol-chloroform-isoamyl alcohol protocol. Briefly, in 600 μl of tissue lysis buffer (0.1M Tris, 0.2M NaCl, 5 mM EDTA, 0.4% SDS, water) and 20 μL of proteinase K (20 mg/ml), the tissue was digested overnight at 50ºC with agitation. To digest any residual RNA, 5 μL RNase A was added and incubated for 30 minutes at 37ºC. DNA was extracted by with phenol-chloroform-isoamyl alcohol and precipitated with the addition of an equal volume of 100% ethanol and 24 μL sodium acetate. The precipitate was resuspended into 40 μL Tris-EDTA. For some tissues, DNA was extracted using Zymo Research Quick-DNA Miniprep Plus kit according to manufacturer's instructions. After extraction, the DNA was quality-checked for RNA contamination using a Qiaxcel Advanced instrument and stored at −20°C. If RNA contamination was present, a Zymo Research DNA Clean and Concentrate kit was used, according to manufacturer's instructions. Mouse organ tissues were processed under the same protocol. Human DNA was obtained from the de-identified “human random control panel 2” (HRC2) series plate derived from lymphoblastoid cell lines by the European Collection of Cell Cultures (purchased from Sigma Aldrich). These individuals ranged from 24–96 years of age. Animals used in this study were maintained in accordance with the Guidelines for the Care and Use of Laboratory Animals (Institute of Laboratory Animal Resources, 1996) and were treated humanely and with regard for alleviation of suffering. The study protocol was approved by the University of Minnesota Institutional Animal Care and Use Committee.

### Global DNA methylation and hydroxymethylation in vertebrates

At least 2 μg of purified genomic DNA from muscle tissue of 54 species and 9 mouse tissues were sent to Zymo Research for analysis. An SRM-based mass spectrometry assay was used to quantify 5-hydroxymethyl-2′-deoxycytidine (5hmdC) and 5-methyl-2′-deoxycytidine (5mdC). The assay was designed to measure 5hmdC concentrations and 5mdC concentrations as a percentage of 2′-deoxyguanosine (dG) [e.g., (5hmdC)/(dG) and (5mdC)/(dG)].

### Ultraconserved noncoding element (UCNE) nomenclature

The database of ultraconserved noncoding elements and genomic regulatory blocks (UCNEbase) provided sequence and nomenclature for 4351 UCNEs used in this study [3]. They are defined as human noncoding regions ≥200 nt and exhibiting ≥95% sequence identity between human and chicken. The nomenclature system was established by UCNEbase for disambiguation of the same conserved element as found in different chromosomal locations in different species. Since UCNEs often cluster near genes, they are named after the putative target gene, e.g., PAX2, and each identified by using common personal names, e.g., PAX2_Daniel, with the personal name alphabetical in order of the element in proximity to the target gene. Singleton UCNEs are referred to by chromosome, e.g., chr11_Kaori. Postscript numbering defines UCNEs with <50 nt separation in human and chicken, e.g., BCL11A_Naoko_2.

### GC and CpG summary statistics

All 4351 UCNEs were downloaded as fasta sequence. GC content was calculated for UCNE group and compared to the 22 autosomes + XY of the human genome. CpG frequency was calculated using a custom Perl script, applied to all UCNE sequences, and also human coding regions (CDS), human introns, and human promoters defined as 1000 nt upstream of coding regions sourced from the UCSC knownGene table of genes using human genome build GRCh/hg38. CpG density is defined as number of CpG sites / length of sequence (or x100 = CpG sites per 100 bp). The single UCNE with highest CpG density, 12.4 per 100 bp (chr1_Aida) was removed from analysis upon discovery that it was misclassified as noncoding. All UCNEs, positions, and CpG density measures can be found in Table S2. T-tests and figures were generated in R version 3.3.3.

### Bisulfite conversion

Fifty-six species were selected for pyrosequencing analysis. DNA was standardized at 200 ng/μl and converted with a Zymo Research 96-well lightning DNA conversion kit according to manufacturer's instructions.

### Primer design and pyrosequencing

All primers were designed using Qiagen Pyromark Assay Design software version 2.0 against the human derived sequence of each UCNE. Primers were optimized for PCR by using bisulfite converted mouse DNA and tested for single-band amplification across all species. The parameters for each reaction included a thermocycler protocol of 95°C for 30 seconds, the optimal temperature for 30 seconds, and 72°C for 30 seconds repeated for 35–40 cycles. Primer sequence, and conditions can be found in Table S1. Conservation values were derived from UCNEbase.

PCR products were quantitated for DNA methylation level on a Qiagen Pyromark Q96 ID instrument. Controls consisted of a “no template control” (NTC) and two wells containing bisulfite converted 100% or 0% methylated control human DNA from EpiGentek. Some sites failed assessment in some species due to species-specific mutations preventing either PCR amplification or pyrosequencing. CpG methylation values from pyrosequencing were collated under criteria that the Pyromark software defined as ‘check’ or ‘passing’, with these values retained for analysis, and discarded if ‘failed’. For mouse tissues, UCNEs were run with 6 biological replicates, 3 males and 3 females. Results were averaged over all replicates, and split by sex. Position 3 in VRK1_Siddhartha failed uniformly in mouse and was removed. Pyrosequencing for the human LINE1 was also performed on the human samples using previously developed primers [42]. We analyzed the CpG sites both individually and by averaging the CpG sites for each UCNE.

### Limitations in UCNE methylation level detection across species

Missing values as seen in Figure 3 indicate the presence of a SNP at the sequencing region, no PCR product, or the failure of pyrosequencing detection. In general, all UCNEs gave strong single band PCR products that did not vary between species. There were several instances where proximal CpG sites differed by over 80% within the same UCNE and were consistent across tissues as seen in mouse; therefore, a mean of DNA methylation across all sites does not capture the variation inherent within these UCNEs.

### Phylogenetic least squares analysis

Phylogenetic signal as measured by Pagel's lambda (λ) was calculated using the average methylation value of all CpG sites per UCNE [48]. The R caper package [49] function ‘pgls’ was used to fit the phylogenetic generalized linear model, investigating the level of methylation against the phylogeny where λ = 0 indicates no phylogenetic signal and λ = 1 indicates strong phylogenetic signal and evolution under the Brownian motion model, using the formula ‘pgls(meth_value) ∼1, lambda = “ML” ’. The phylogenetic tree for the species used was generated from http://timetree.org with branch lengths based on evolutionary time scales derived from multiple publications [50].

## Supplementary Material

supp_data_1411447.zip
